# Longitudinal quality of life analysis in a phenylketonuria cohort provided sapropterin dihydrochloride

**DOI:** 10.1186/1477-7525-11-218

**Published:** 2013-12-30

**Authors:** Teresa D Douglas, Usha Ramakrishnan, Julie A Kable, Rani H Singh

**Affiliations:** 1 Metabolic Nutrition Program, Division of Medical Genetics, Emory Department of Human Genetics, 2165 North Decatur Road, Decatur, GA 30033, USA; 2 Nutrition and Health Sciences Program, Division of Biological and Biomedical Sciences, Laney Graduate School, Emory University, Atlanta, GA USA; 3 Hubert Department of Global Health, Emory University Rollins School of Public Health, 1518 Clifton Road NE. Room 7009, Atlanta, GA 30322, USA; 4 Fetal Alcohol Syndrome and Drug Exposure Center, Marcus Autism Center, 1920 Briarcliff Road, Atlanta, GA 30329, USA; 5 Division of Autism and Related Disorders, Department of Pediatrics, Emory University School of Medicine, Atlanta GA, USA

**Keywords:** Phenylketonuria, PKU, Quality of life, Sapropterin, Tetrahydrobiopterin, BH4, Medical diet

## Abstract

**Background:**

Sapropterin dihydrochloride effectively lowers plasma phenylalanine (Phe) for at least a third of phenylketonuria (PKU) patients, with potential for increased dietary Phe tolerance and decreased medical food requirement.

**Objective:**

To investigate long-term quality of life (QOL) in patients with phenylketonuria (PKU) who took sapropterin (BH4, Kuvan®) for up to one year.

**Methods:**

37 PKU patients, ages 10–49 years, were asked to complete a PKU-specific self-report QOL questionnaire (QOLQ) at baseline, 1, 4, 8, and 12 months. Questions were scored on a 5-point Likert scale under 5 sub-sections measuring Impact, Worries, Satisfaction, Support, and General wellbeing in relation to PKU. Responders with a plasma Phe decrease ≥ 15% after 1 month on sapropterin remained on the drug; Nonresponders ceased sapropterin after the trial month. Responders able to relax medical diet and maintain plasma Phe control were classified as Definitive; Responders unable to relax medical diet were classified as Provisional. All patients were routinely monitored by a registered dietitian. Data was analyzed in SPSS 19.0 using regression techniques.

**Results:**

Of 17 Responders, 11 could maintain adequate Phe control on a less restrictive diet. One year mean Impact sub-score trends improved significantly for all sapropterin response groups, with greatest improvement among Definitive Responders (*p* < 0.0001). Satisfaction sub-scores also improved for Definitive Responders (*p* = 0.001). Trends for Total QOL score improved significantly over time for both Definitive (*p* = 0.001) and Provisional Responders (*p* = 0.028). Improvements in Definitive Responder scores were associated with increased Phe tolerance (Impact: *p* < 0.0001, Satisfaction: *p* = 0.022, Total QOL: *p* = 0.005) and MF adjustment (Satisfaction: *p* = 0.014, Total QOL: *p* = 0.026). Other sub-section scores remained steady, unaffected by sapropterin response or diet modification.

**Conclusion:**

Increased Phe tolerance and reduced MF requirement in sapropterin Definitive Responders improves QOL perception across one year, specifically for life impact and satisfaction.

## Background

Phenylketonuria (PKU) is a recessively inherited inborn error of metabolism. Impaired phenylalanine hydroxylase (PAH) activity in PKU inhibits the effective metabolism of its phenylalanine (Phe) substrate. Blood Phe concentrations can therefore increase to neurotoxic concentrations. Since Phe is ubiquitously found in most food sources, to prevent neurologic damage induced by high Phe concentrations, those with PKU must follow a strict low Phe diet throughout their lives and consume a Phe-free amino acid rich medical food as their primary source of protein nutrition. This diet treatment strategy involves daily monitoring of medical food and Phe intake so that patients with PKU can maintain plasma Phe concentrations within the safe therapeutic threshold of 100–360 μmoles/L.

Although newborn screening has enabled dietary treatment for PKU to begin during early infancy, thereby avoiding neurologic consequences such as severe lifelong developmental and cognitive disability, the dietary regimen for PKU can be rigorous, expensive, and socially burdensome to both families and patients [1,2]. In particular, patients transitioning into adolescence and adulthood will demonstrate decreased dietary compliance and plasma Phe concentrations increased beyond the therapeutic range [3,4]. Although the consequences are not as severe as during infancy and young childhood, this noncompliance has been shown to lead to neurological sequelae such as increased risk of mood disorders [5], attention deficits [6,7], impairments to executive functioning [8-10], school performance and achievement [11], and social difficulties [12], issues which can certainly impact QOL. Past studies have implicated both high plasma Phe concentrations as well as the strict medical diet in reduced quality of life (QOL) for patients with PKU [13,14].

Sapropterin dihydrochloride, a pharmaceutical form of the chaperone PAH cofactor tetrahydrobiopterin (BH4), not only lowers plasma Phe concentrations for up to half of patients with PKU [15], but many of these sapropterin responders are reported to also experience notable increases to dietary Phe tolerance, allowing for a less restricted diet as long as they remain on the pharmaceutical [16]. This study is the first to evaluate QOL in a combined adolescent and adult cohort of patients with PKU for one year after initiating sapropterin. Our objective was to determine if decreased plasma Phe and increased dietary Phe tolerance induced by sapropterin would improve self-reported QOL in adolescent and adult patients with PKU.

## Methods

### Study design and patient enrollment

Patients 10 years of age and older with PKU who planned to start sapropterin dihydrochloride (Kuvan, BioMarin Pharmaceutical Inc.) were asked to volunteer for a 1 year prospective cohort study in which QOL would be evaluated in the form of a self-report questionnaire. All patients were recruited through the Emory University Genetics Clinic in Atlanta Georgia. Enrollment lasted from October 2008 through October 2009. Participants were asked to complete the questionnaire prior to starting sapropterin as well as at 1, 4, 8, and 12 months thereafter. Blood was collected during corresponding clinic visits for evaluation of plasma amino acids (PAA) (Biochrom 30 HPLC Amino Acid Analyzer) (μmoles/L). Three day food records were collected and analyzed by a licensed registered dietitian. Food records were analyzed using Nutrition Data System for Research (NDSR) to calculate average daily intake of energy (kcals), Phe (mg), and protein (g). Reported intake of medical food protein and dietary Phe was assessed in parallel with the patients’ medical food protein prescription (g/day) and dietary Phe tolerance (mg/day), with the dietitian adjusting as needed.

Preliminary response to sapropterin was determined after one month of taking 20 mg/kg/day of the medication. Patients were instructed to continue with current medical food and dietary Phe practices to ensure that observed changes in plasma Phe were due to sapropterin response rather than dietary manipulation. Patients were classified as Preliminary Responders if plasma Phe decreased by at least 15% and continued on sapropterin throughout follow-up. Patients without this decline in plasma Phe were classified as Non-responders and discontinued the medication. All patients met with a registered dietitian during clinic visits. Non-responders were encouraged to continue with their prescription diet plan, while Preliminary Responders were provided Phe challenge and assessed for decreased medical food requirement. In agreement with criteria published by Singh and Quirck [16], Preliminary Responders who could maintain plasma Phe below the 360 umol/L therapeutic threshold while increasing Phe tolerance and decreasing medical food intake were determined to be Definitive whereas Provisional Responders could not increase Phe or lower medical food without compromising plasma Phe.

Patients provided written informed consent, or the legal guardian’s written informed consent with age appropriate patient verbal or written assent, prior to study participation. Study protocol and informed consent procedures were approved by the Emory University institutional review board (IRB). Inclusion criteria were a diagnosis of PKU, minimum age of 10 years, and intent to begin sapropterin as a treatment for PKU. Exclusion criteria were having had biopterin therapy within 8 weeks prior to study involvement, pregnancy or planning to become pregnant, and literacy or comprehension difficulties that would limit patient ability to provide informed consent or complete the QOL questionnaire.

### Quality of life questionnaire

The QOL questionnaire selected for this investigation, the PKU-QOLQ, was developed to evaluate QOL parameters specific to the experience of living with PKU. The PKU-QOLQ is patterned after the validated juvenile diabetes QOL questionnaire [17] and has been shown to predict positive adaptive outcome in a cohort of adolescent females with PKU [18]. The adapted PKU-QOLQ consists of five quantitative subsections, each with a subset of numbered questions that can be answered on a five-point Likert scale (subsections and scoring ranges are described in Table 1). Subsections evaluated 5 areas in which PKU could influence quality of life: impact of PKU on life quality, PKU related life worries, satisfaction with life and medical management, sense of support from social network and clinical community, and perspective on general well-being. For Impact and Worries, 1–5 choices were “all the time, often, sometimes, very seldom, and never”. For Satisfaction and General sections, 1–5 Likert scale choices were “very unsatisfied, somewhat unsatisfied, neither, somewhat satisfied, very satisfied”. Subscores for the quantitative subsections were combined into a total QOL score.

**Table 1:** Baseline PKU-QOL total score and sub-score characteristics

				***QOL Scoring***	
	**Mean ± SD**	**Range**	**n scores > mid-score**	***Min-Max***	***Mid-score***
**Scoring total (n = 34)**	**206 ± 29**	**109 to 246**	**32**	***51-255***	***153***
Impact (n = 35)	71 ± 11	31 to 85	32	*18-90*	*54*
Worries (n = 35)	44 ± 8	13 to 50	33	*10-50*	*30*
Satisfaction (n = 35)	30 ± 7	15 to 40	26	*8-40*	*24*
Support (n = 34)	18 ± 3	10 to 20	30	*4-20*	*12*
General well-being (n = 34)	44 ± 8	30 to 54	29	*11-55*	*33*

n is the number of returned questionnaires with completed sections at baseline.

Minimum score: Lowest score possible if all answers in section scored a 1. Maximum score: Highest score possible if all answers in section scored a 5. Mid-score: If all answers in a section scored a 3.

### Statistical analysis

Study sample size was determined a-priori using SPSS SamplePower 2.0 software. SamplePower’s sample size and power analysis technique utilize Cohen’s calculations for determining effect size and study power [19]. For this investigation, an effect size of 1.22 was calculated from an r^2^value of 0.55 found in a 2008 paper which showed association between diet related plasma Phe improvements and quality of life in PKU patients [13]. Thus, for this study, a minimum sample size of 18 was determined to be sufficient for a power of 0.8 with α = 0.05, and a maximum of 5 covariates in linear models.

Statistical analysis of final data was completed using SPSS 19.0. Baseline analysis of variable associations with QOL scores utilized Pearson correlation and multivariate linear regression. Cox regression survival analysis was used to investigate the impact of baseline variables on attrition. ANCOVA by generalized linear method was used for analysis across groups between baseline and one month. Mixed linear regression using an unstructured variance-covariance type model was used to evaluate trends across one year of follow-up since the method is robust in circumstances of unbalanced longitudinal data [20]. When relevant, mixed models were adjusted for significant variation in intercepts or slopes. Variables evaluated for association with QOL outcomes include age, gender, physical activity level (scored on a 1–5 Likert scale of low to high physical activity), income, education, family marital status, prescribed medical food protein, dietary Phe tolerance, plasma amino acids, and sapropterin response. All analyses were two-tailed with α = 0.05.

## Results

### Demographics, sapropterin response, and attrition

Thirty-seven male and female patients, age 10–49 years (mean ± SD: 22.1 ± 9.4) enrolled in the study. Baseline demographic data is portrayed in Table 2. At one month, 36 patients returned, with 17 classified as preliminary Responders and 19 as Non-responders. Of the preliminary Responders, Phe challenge revealed 11 to be Definitive and 6 to be Provisional by the study’s fourth month. Seven of the Non-responders were lost to follow-up before the study year was complete. Support sub-scores at baseline, but no other QOL outcomes, had a positive correlation with length of time Non-responders remained in the study (r = 0.45, *p* = 0.005). Single marital status was also associated with higher attrition in Non-responders (*p* = 0.037). Plasma Phe, dietary Phe, medical food intake, age, gender, income, and education level were not associated with attrition. Of the 29 patients who completed the long-term QOL study, 8 failed to return the PKU-QOLQ one or more times during the study while 2 others did not complete all subsections of the PKU-QOLQ on one or more occasions.

### QOL at baseline

Table 1 describes the average QOL scores, and scoring ranges, for the PKU cohort at baseline. Total and sub-scores averaged high, with at least 74% of participants having baseline sub-scores above the mid-score and 94% having a total QOL score above mid-score.

**Table 2:** Baseline demographics of study participants (N = 37)

**Variable**	**N (%)**
**Gender**	**--**
Male	20 (54%)
Female	17 (46%)
**Age (years)**	**--**
Adolescent (10–19)	19 (51%)
Adult (20+)	18 (49%)
**3 pairs of biological siblings**	6 (16%)
**Neuropsychiatric and Behavioral diagnoses**	**--**
No diagnosis	29 (78%)
ADHD	4 (11%)
Other (Depression, Bipolar, Anxiety)	4 (11%)
**Family marital status‡**	**--**
Single	7 (19%)
Married	26 (70%)
Divorced	3 (8%)
Widowed	1 (3%)

‡Marital status represents participants themselves age 19+, and of the legal guardians for patients <19 years.

**Figure 1:**
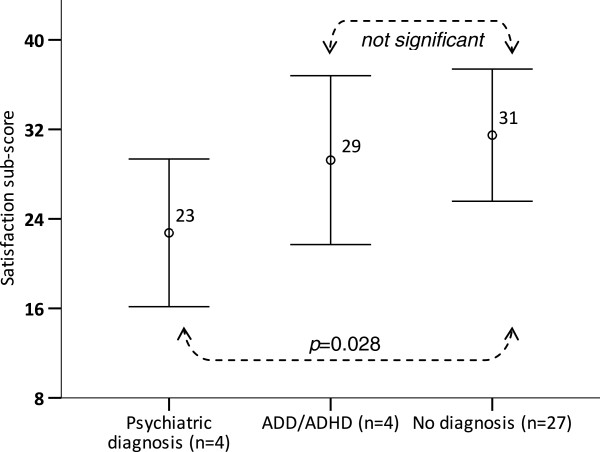
**Baseline Satisfaction sub-scores (mean ± 1 SD) across PKU patients with and without psychiatric diagnosis (Depression, Anxiety, Bipolar) and ADHD.***P*-values are Bonferroni adjusted for multiple comparisons.

Total QOL score and all sub-scores other than Support sub-score were inversely correlated with age (Table 3); associations with QOL scores, except for Support sub-score, are therefore age adjusted. Plasma tyrosine was inversely associated with Worries sub-score (*p* = 0.025) at baseline but with no other QOL scores. Self reported physical activity level had a positive correlation with General well-being sub-score (*p* = 0.018), while patients diagnosed with one or more psychiatric disorders (ie: depression, anxiety, bipolar disorder) reported lower Satisfaction scores (*p* = 0.028) (Figure 1). Plasma Phe, prescribed diet, marital status, income and education level were not associated with baseline QOL scores. There were also no differences in baseline PKU-QOL scores among prospective sapropterin response groups. Controlling for same-household siblings did not affect results.

**Table 3:** Baseline age-adjusted correlations of PKU-QOL scores

	**Impact**	**Worries**	**Satisfaction**	**Support**	**Generally**	**Total score**
Age (years)	-.37*	-.35*	-.48**	NS	-.55**	-.58***
	(−.63, -.04)	(−.61, -.02)	(−.70, -.17)		(−.75, -.26)	(−.77, -.30)
Plasma Phe (μmoles/L)	NS	NS	NS	NS	NS	NS
Plasma Tyrosine (μmoles/L)	NS	-.39*	NS	NS	NS	NS
		(.07, .64)				
Phe tolerance (mg/day)	NS	NS	NS	NS	NS	NS
MF protein Rx (g/day)	NS	NS	NS	NS	NS	NS
Physical activity	NS	NS	NS	NS	.41*	NS
					(.08, .66)	
Psych/Behavior diagnosis‡	NS	NS	-.38*	NS	NS	NS
			(−.63, -.05)			

Pearson coefficient (95% CI).

*P < .05, **P < .01, ***P < .001.

CI = Confidence Interval, Rx = Prescription, MF = Medical food.

NS = Not statistically significant.

‡: Psychological and behavior diagnosis dummy coded in SPSS: 1 = no diagnosis, 2 = ADHD diagnosis, 3 = psychiatric diagnosis (Bipolar, Depression, Anxiety).

### QOL after 1 month of sapropterin

**Figure 2:**
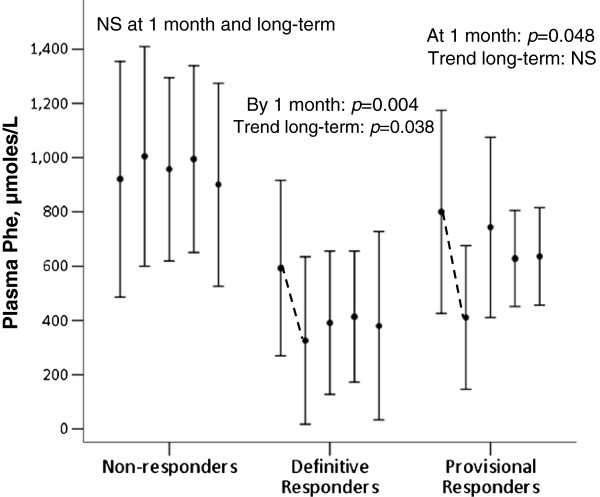
**Plasma Phe (mean ± 1 SD) of sapropterin response groups across 5 study visits.** Clustered bars represent study visits at Baseline, 1, 4, 8, and 12 months respectively. NS = Not significant.

Characteristic of preliminary response to sapropterin, plasma Phe significantly dropped (*p* = 0.008) after the first month for 47% of study patients (Figure 2). However, no statistically significant change in PKU-QOL scores occurred within or between Responders and Non-responders from baseline to 1 month, even when controlling for later determined Definitive and Provisional response classification.

### QOL during 1 year

Baseline associations of age and physical activity with QOL scores were unaltered in the long-term analysis and were thus controlled for in relevant mixed regression models. Although plasma tyrosine was inversely associated with Worries sub-scores at baseline, the association was not significant long-term regardless of sapropterin response classification. The impact of psychiatric or ADHD status on long-term QOL outcomes could not be determined due to the attrition within those smaller categories.

**Figure 3:**
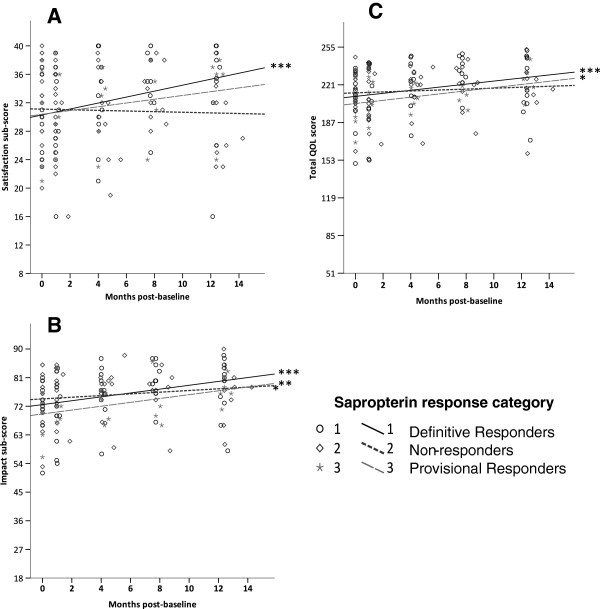
**(A,B,C): Impact and Satisfaction sub-score (A and B) and total QOL (C) trends across one year.** *p < 0.05, **p < 0.01, ***p < 0.001. *p*-values for longitudinal trends are age adjusted. Y-axis begins at minimum score.

There were no statistically significant differences between sapropterin response groups in PKU-QOL scores during 1 year of follow-up, however significant within group changes did occur. All response groups demonstrated a trend towards improved Impact sub-scores, with Definitive Responders experiencing the greatest improvement (*p* < 0.0001) compared to Provisional Responders (*p* = 0.01) and Non-responders (*p* = 0.05) (Figure 3B). Trends for Satisfaction sub-scores increased significantly for Definitive Responders only (*p* = 0.001) (Figure 3A), while Worries, Support, and General well-being sub-scores did not change significantly, irrespective of sapropterin response. Trends in Total QOL scores significantly improved for both Definitive (*p* = 0.001) and Provisional Responders (*p* = 0.028) (Figure 3C). Although Non-responders experienced a modest improved trend in Impact sub-scores, total QOL scores for Non-responders did not change significantly over one year.

### Association of plasma Phe, Phe tolerance, and prescribed medical food with long-term QOL scores

Table 4 details the significance of regressed associations between long-term QOL scores and the modifiers plasma Phe, dietary Phe tolerance, and prescribed medical food protein.

**Table 4:** **Variable interactions**^‡^**with QOL scores across time for sapropterin response groups and whole study sample †**

	**Impact**				**Satisfaction**				**Total score**			
	**D**	**N**	**P**	**All**	**D**	**N**	**P**	**All**	**D**	**N**	**P**	**All**
Phe tolerance (mg/day)	(+)***	(+)*	NS	(+)***	(+)*	NS	NS	(+)*	(+)**	NS	NS	(+)*
MF protein Rx (g/day)	NS	NS	NS	(−)*	(−)*	NS	NS	(−)*	(−)*	NS	NS	NS
Plasma Phe (μmoles/L)	(+)**	(+)*	NS	(+)**	(+)***	NS	NS	(+)**	(+)**	NS	NS	(+)*

*p < 0.05, **p < 0.01, ***p < 0.001.

‡Interaction defined as “variable*time*response category” in longitudinal mixed regression analysis.

†Results for Worries, Support, and Generally sub-scores not included since no significant changes occurred across time for those outcomes.

(+) indicates positive association, (−) indicates negative association. NS = not statistically significant.

**D**: Definitive Responders, **N**: Non-responders, **P**: Provisional Responders, **All**: Complete study sample.

Significant long-term declines in plasma Phe among Definitive Responders (*p* = 0.002) (Figure 2) was associated with linear increases to Satisfaction (*p* < 0.0001), Impact (*p* = 0.002), and total QOL scores (*p* = 0.001). Interestingly, plasma Phe had a direct association with Impact sub-scores in Non-responders (*p* = 0.038), though was not associated with Provisional Responder’s QOL scores.

**Figure 4:**
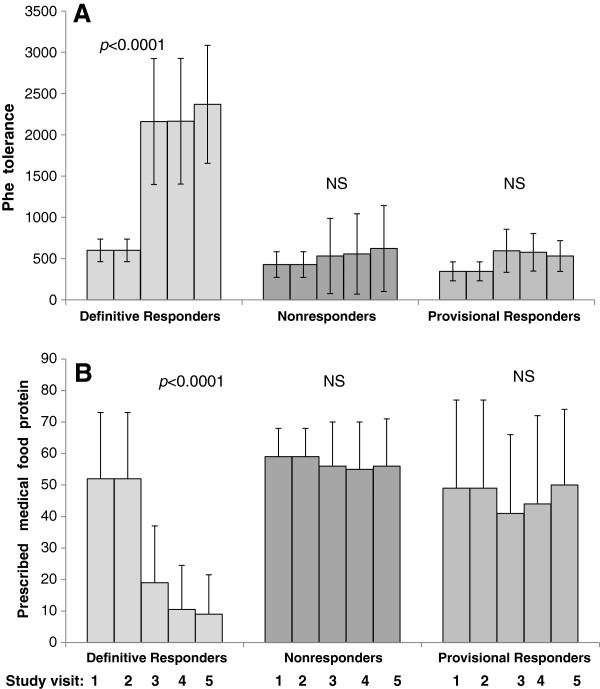
**(A and B): Mean (± 1 SD) Phe tolerance (mg/day) and prescribed medical food protein (g/day) of sapropterin response groups across study visits.** Clustered bars represent study visits at Baseline, 1, 4, 8, and 12 months respectively. NS = Not significant.

Observed increases to dietary Phe tolerance inherent to Definitive Responder status (Figure 4A) were associated with improved trends in Impact (*p* < 0.0001), Satisfaction (*p* = 0.025), and Total (*p* = 0.005) QOL scores across one year. Phe tolerance, remaining unchanged for Provisionals and Non-responders, had a significant positive association with Impact subscores for Non-responders (*p* = 0.012), although not for Provisional’s (*p* = 0.062).

Significant reductions to prescribed medical food protein (g/day) for Definitive Responders (Baseline Mean ± SD: 51.8 ± 20.8, 1 year: 9.0 ± 12.5) (*p* < 0.0001) (Figure 4B) accommodated increases to protein and energy intake from intact food sources within the limits of effective Phe control. Reductions to medical food Rx in Definitive Responders was associated with better Satisfaction sub-scores (*p* = 0.014) and total QOL score (*p* = 0.026). For the undifferentiated PKU cohort, higher Impact scores had a long-term linear association with lower medical food prescriptions (*p* = 0.04), but the association was not maintained once stratified by sapropterin response groups. No other QOL scores were influenced by medical food prescription long-term, irrespective of sapropterin response.

## Discussion and conclusion

**Figure 5:**
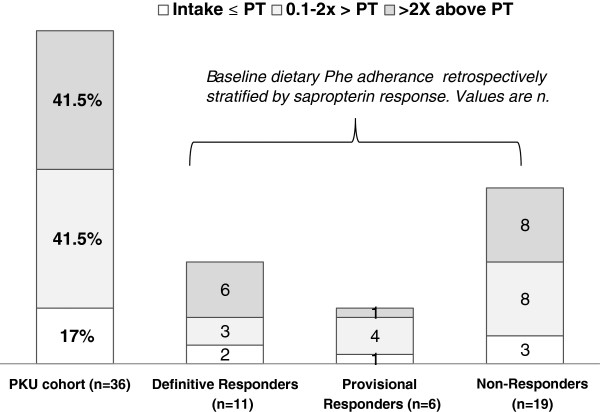
**Baseline dietary Phe compliance of patients with PKU.** ‡Dietary Phe compliance defined as patient-reported Phe intake in excess of Phe tolerance (PT) (mg/day). One patient with undetermined baseline Phe tolerance omitted from figure.

The observation that most of our study patients had QOL scores exceeding the mid-score, with high means at both baseline and throughout the study (Table 1, Figures 3A-C), is consistent with other research demonstrating good QOL in adults, adolescents, and young children with PKU when compared to non-PKU reference samples [12,21-23], although Cotungo’s 2011 study did discover lower parent reported QOL scores for children with PKU compared to a non-PKU reference group [21]. Since the focus of our study was to investigate sapropterin’s influence, along with relevant dietary modifications, on self-reported QOL within a cohort living with PKU, as opposed to comparing QOL in PKU to that of non-PKU populations, our results do not conflict with the Cotungo’s 2011 pediatric results. The high baseline scores of our study patients, however, may create a ceiling effect that inhibits observation of long-term changes; in essence, improvements to quality of life may not be as measurable when quality of life is already near the upper scale limits. This phenomenon may be a reflection of patient adaptation, in an emotional and psychological sense, to their disorder [24,25] or it may indicate a need for improved sensitivity in the questionnaire’s QOL scale. In addition, 83% of the cohort reported Phe intake above Phe tolerance at baseline (Figure 5); thus, improvements to QOL extending from sapropterin induced increases to Phe tolerance could be attenuated in patients already consuming liberal amounts of dietary Phe. This may explain the lack of observed change in the PKU-QOL sub-scores for Worries, Support, and General well-being.

Even so, we were able to detect improvements in Impact, Satisfaction, and Total QOL outcomes across one year in our patient sample, most pronounced in those PKU patients on sapropterin able to liberalize diet. This may seem contrary to Ziesch’s study where no changes in health related QOL were observed after 3 months in pediatric patients with PKU provided sapropterin, irrespective of ability to liberalize diet while maintaining good blood Phe control. However, our analysis of a larger adolescent & adult cohort evaluated QOL multiple times across a full year, allowing us to observe the gradual long-term positive direction of Total, Impact, and Satisfaction scores. These beneficial outcomes were particularly evident in Definitive Responders. Of important distinction also is our use of a questionnaire tool specifically developed for measuring QOL in individuals with PKU. A lack of available QOL tools specific to inborn errors diagnoses was acknowledged by Ziesch as a possible limitation in his QOL study [22]. Although the PKU-QOLQ is still in the process of validation for males and other age groups, it proved highly effective at assessing QOL in our study sample, when controlling for age, with no observed gender differences in scoring outcomes. Internal consistency was considered good, with Cronbach Alpha > 0.8 for the questionnaire and its subsections, as well as across age and gender groups (Table 5). Hence, the differences in our study design, tools, and patient cohort offers the best explanation for the discrepancy between our study and Ziesch’s.

**Table 5:** Cronbach Alpha analysis of PKU QOL questionnaire

	**No. of questions**	**Overall alpha**	**Alpha for adults**	**Alpha for pediatrics**	**Alpha for males**	**Alpha for females**
Impact	18	0.87	0.84	0.89	0.89	0.85
Worries	10	0.91	0.86	0.94	0.93	0.86
Satisfaction	8	0.88	0.87	0.89	0.90	0.86
Support	4	0.88	0.91	0.90	0.92	0.87
General well-being	11	0.90	0.90	0.93	0.91	0.92
**Total questionnaire**	**51**	**0.95**	**0.94**	**0.96**	**0.96**	**0.94**

An interesting observation in this analysis was that differences in Phe tolerance were not associated with QOL outcomes at baseline, yet increased Phe tolerance over the course of one year in Definitive Responders was significantly associated with improved QOL outcomes. The lack of baseline association may be due to the psycho-emotional adaptation of these patients to the constraints of PKU diagnosis, as discussed earlier, particularly since patients have lived with the condition since birth. However, a fast dramatic increase in Phe tolerance that is sustained as a result of sapropterin treatment could boost QOL self-perception when measured over time. Whether that improvement in QOL can be sustained, or whether patients will habituate to their higher Phe tolerance with QOL gradually returning to baseline, remains to be seen in future analysis where follow-up extends beyond one year.

Also noteworthy is that Non-responders and Provisional Responders were without significant change to Phe tolerance or formula dependence, yet had significant improvement to their Impact QOL scores. The trend seen in Provisionals and Non-responders was modest compared to the improved QOL observed in Definitive Responders, but being statistically significant still deserves explanation. The modest QOL increase in these two groups may be due to a Hawthorne effect: essentially, the patients’ answers to QOL questions improved over time as a result of monitoring within the study parameters. Additionally, the QOL scores could reflect patient emotional benefit to increases in medical surveillance and monitoring, since all study subjects received more frequent personalized medical attention throughout the study year. A placebo effect in the case of Provisional Responders is also possible. Patients within this group anecdotally reported the benefit they felt sapropterin had on their lives even though their diets were not significantly liberalized. Therefore, patient self-reports of life benefits and improved emotional outcomes while on sapropterin should be considered objectively when these patients meet the criteria for provisional response [16]. If while on sapropterin patients are not benefitting from improved Phe control, nor demonstrating improved Phe tolerance, the clinical benefit of sapropterin in their PKU management plan should be scrutinized, particularly since impact on QOL would most likely be minimal.

Study results indicate baseline Support scores, in addition to other factors such as marital status and sapropterin response classification, could effectively predict long-term attrition within our PKU study cohort. In fact, sense of emotional support had the strongest relationship with study attrition, such that the amount of weeks spent in the study could be predicted by how high the support scores were at baseline, particularly in Non-responders where most of the attrition occurred. Interestingly, the Support section represents the smallest question cluster in the QOLQ. There are only 4 queries which ask about support received from the person’s family, friends, medical team, and nutrition team. An improvement in just one or two of these support aspects could dramatically reduce attrition risk in future studies, thus it may be worthwhile to ensure patients are provided an emotionally supportive clinical environment from their very first appointment when long term follow-up is indicated.

In agreement with other QOL studies, our analysis revealed an inverse association between age and QOL scores [26-28], thereby stressing the need to control for age when evaluating QOL differences in clinical groups, or to incorporate an age diversified QOL questionnaire system. Physical activity should also be controlled for when evaluating QOL, as our study indicated.

Due to several adult PKU study patients being neither employed nor in school, two work/school questions were frequently skipped. Modifications to the PKU-QOLQ may be necessary to accommodate this circumstance in adults, however for this study we resolved the issue by eliminating those two specific questions from final scoring and analyses.

Our study focused on patient self-reported QOL only; however, improved QOL outcomes may also occur in parents, caregivers, spouses, or other family members when an individual with PKU is able to increase dietary freedom due to a new treatment, such as sapropterin. Though our clinicians have received many positive anecdotal reports from family members and caregivers of sapropterin Responders (both Definitive and Provisional), a formal analysis is needed to determine measurable QOL benefits to these family members and caregivers. Such an analysis was beyond the scope of this investigation but would be a worthwhile pursuit for future study.

The present study results provide additional opportunity to assist patients with PKU in areas where they report feeling most challenged or discouraged from a social-medical perspective. The information gleaned can assist with technicalities of adjusting therapeutic protocols as patients gain access to emerging treatment options, such as sapropterin, which may offer more dietary freedom, as well as with planning of long term clinical studies. In this respect, patients can receive the emotional, medical, and nutritional support they need for effective lifelong management of their disorder.

## Conclusion

Self-reported quality of life measured high on average in adolescents and adults with PKU. Nevertheless, marked improvements occurred in the QOL areas where impact on various aspects of life and self-sense of satisfaction were measured. Improvements were most dramatic for patients who experienced increased Phe tolerance while taking sapropterin. These improvements were accomplished while maintaining good Phe control in the majority of Definitive Responders. Non-responders and Provisionals, who had no increase in Phe tolerance, still demonstrated modest improvements to QOL during the study, albeit to a lesser extent than Definitive Responders. Aspects of general well being, sense of social support, and worries in relation to PKU and life were not associated with sapropterin response classification, Phe tolerance, medical food prescription, or PAA during the long-term study.

## Abbreviations

PKU: Phenylketonuria; PAA: Plasma amino acids; QOL: Quality of life; QOLQ: Quality of life questionnaire; BH4: Tetrahydrobiopterin: sapropterin.

## Competing interests

Independent Investigator sponsored trial with partial funding provided by BioMarin Pharmaceutical Inc.

## Authors’ contributions

TDD was responsible for grant and protocol writing, adaptation of QOLQ for adult study population, distribution and collection of questionnaires, data entry, data management and analysis, statistical interpretation, and writing all parts of the manuscript including tables and graphs. UR made significant contributions in reviewing the article, advising analytical and statistical approaches as well as appropriate data management techniques, in recommending large scale revisions, and for providing additional project guidance and support. JAK provided crucial guidance regarding data collection, management, and analysis; and reviewed data results, provided feedback on statistical approaches, and assisted with reviewing and editing of the article drafts. RHS was the study Principle Investigator, provided management and supervision of grant writing, protocol development, patient dietetic management, data management, staff research activities, and data reporting; contributed to review and revising all manuscript drafts. All authors read and approved the final manuscript.
